# Cardiac Arrest During Spinal Surgery: A Multidisciplinary Approach and Management of Takotsubo Syndrome

**DOI:** 10.7759/cureus.32404

**Published:** 2022-12-11

**Authors:** Mafalda Silva, Noélia Carrillo-Alfonso, Pedro Amorim

**Affiliations:** 1 Anestesiology, Centro Hospitalar Vila Nova de Gaia/Espinho, Porto, PRT; 2 Anesthesiology, Centro Hospitalar Universitário do Algarve, Faro, PRT; 3 Anaesthesia, Centro Hospitalar Universitário do Porto, Porto, PRT

**Keywords:** prone position, neurosurgery, takotsubo syndrome, intraoperative monitoring, cardiac arrest

## Abstract

Cardiac arrest (CA) in the operating room is rare. Among known perioperative CA causes, Takotsubo syndrome (TTS) is a well-recognized one. Perioperative TTS is more common than existing reported cases therefore anesthesiologists should be aware of its diagnosis and management. TTS is an acquired and self-limited stress cardiomyopathy usually triggered by a precipitating stress factor and should be considered in any hospitalized patient presenting with symptoms such as acute coronary syndrome, cardiac arrhythmias or CA. A 67-year-old woman presented for lumbar discectomy in knee-chest position suffered CA two hours after the beginning of surgery. After a thorough examination, TTS was determined as the cause. This case report highlights the importance of TTS as a differential diagnosis of CA in the intraoperative setting as well as the usefulness of multimodal monitoring with cerebral monitoring to assist the prompt pulseless electric activity diagnosis. To our knowledge, this is the first case report in such circumstances.

## Introduction

Cardiac arrest (CA) in the operating room is a rare and potentially fatal event [1]. Among known perioperative CA causes, Takotsubo syndrome (TTS) is a well-recognized one [2]. TTS is an acquired and self-limited stress cardiomyopathy with an estimated incidence of 2-9/100,000 people annually and may occur in up to one in 6,700 cases in the perioperative period; therefore, anesthesiologists should be aware of this syndrome as well as its diagnosis and management [3,4].

TTS occurs more frequently in females, in post-menopausal age, and is usually triggered by a precipitating stress factor. Clinically, it mimics an acute coronary syndrome (ACS), but coronaries are normal on cardiac catheterization. It is likely caused by excessive catecholamine stimulation although the exact etiology is still under discussion [3,5,6]. In the intraoperative setting, it can be triggered by the surgical condition, the surgical procedure, or the anesthesia itself [3].

Diagnosis is made by observation of left ventricular apical ballooning in echocardiogram and ventriculography [5,7]. Treatment is frequently supportive aiming at minimizing potential complications during spontaneous recovery [4]. Prognosis is usually good with more than 90% of patients surviving the acute episode [3].

We report a case of a CA secondary to TTS in a patient undergoing spine surgery in the knee-chest position. The event occurred two hours after the surgical incision without any apparent cause. We aim with this case report to highlight that TTS should be considered a differential diagnosis of CA in the intraoperative setting in any patient presenting with ACS, hemodynamic instability or cardiac arrhythmia and CA. We aim as well to emphasize the use of multimodal monitoring with cerebral monitoring to assist the prompt pulseless electric activity (PEA) diagnosis. Signed informed consent was obtained from the patient after resolution of an acute event.

## Case presentation

A 67-year-old woman presented for lumbar discectomy. The previous history included osteoporosis, and severe depression, treated with clonazepam, lorazepam and clomipramine. She previously underwent three minor surgeries without complications. Her body mass index was 27 kg/m^2^. She had a functional capacity of > 4 METS and had no cardiovascular disease. The preoperative evaluation was normal.

Upon arrival in the operating room, the patient was extremely anxious. Vital signs were within the normal range. Monitoring included SpO_2_, 5-lead ECG, non-invasive blood pressure (NIBP), neuromuscular blockade, temperature, bilateral density spectral array (DSA) with Bispectral Index™ (BIS), regional cerebral oxygen saturation (rSO_2_) with unilateral INVOS™5100 and Analgesia Nociception Index (ANI).

Remifentanil and propofol by target-controlled infusion (TCI) were initiated, followed by intravenous (IV) lidocaine and rocuronium. Deep neuromuscular blockade was maintained with rocuronium perfusion. Remifentanil was titrated according to ANI during surgery. BIS values were kept within 40-60.

**Figure 1:**
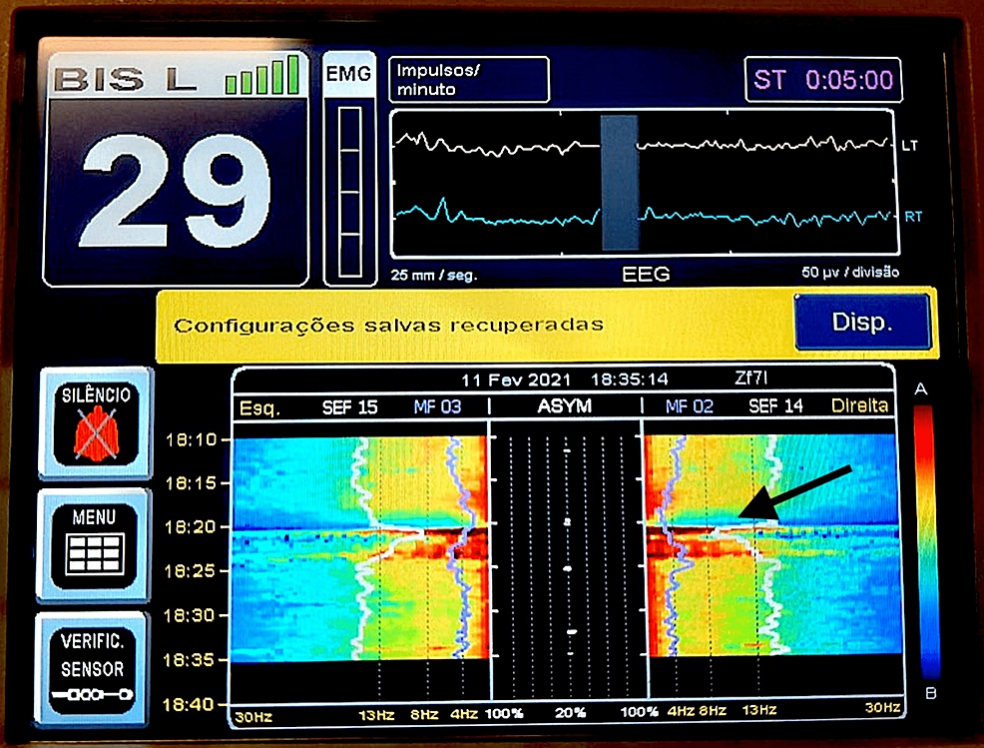
Electroencephalography (EEG) spectrum during pulseless electric activity (PEA) Bispectral index (BIS) monitors image measuring EEG spectrum during PEA due to Takotsubo syndrome (TTS). The arrow shows a bilateral decrease of all frequency power on color density spectral array (DSA) during cardiac arrest (CA) in PEA, followed by an increase of all frequencies power after the return of spontaneous circulation (ROSC) and brain reperfusion. Succeeding DSA is compatible with a general anesthesia state, with a similar spectral edge frequency (SEF) tendency before CA. No relevant asymmetry was registered.

**Figure 2:**
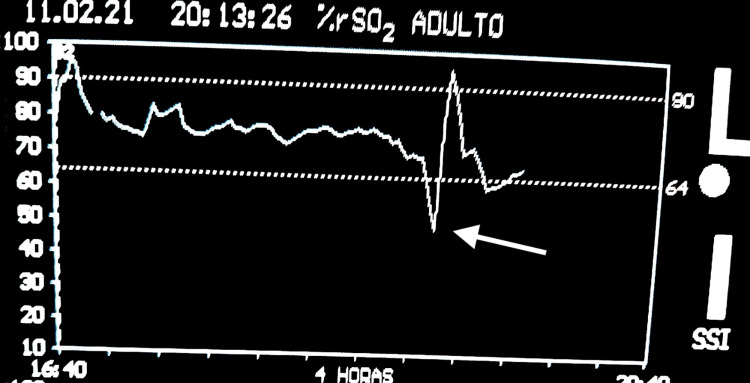
Regional cerebral oxygenation (rSO2) measured with the INVOS monitor INVOS monitor image measuring rSO_2_ during pulseless electric activity (PEA). Arrow shows sudden decrease of cerebral oxygenation after PEA followed by a large rSO_2_ increase due to brain reperfusion as a consequence of return of spontaneous circulation (ROSC).

Before and after placement in the knee-chest position all variables monitored remained within normal limits. BP was permissively low, without compromise neither of rSO_2_ nor the ST-segment. Surgery was technically a challenge as the hernia was difficult to access, and a minor durotomy with cerebrospinal fluid (CSF) leakage occurred. Two hours after the beginning of surgery 200 mL of blood were lost over a period of 15 minutes and ephedrine 30 mg and atropine 0.5 mg were administered to maintain BP. A few minutes later pulse oximetry became undetectable and BP reading unmeasurable, while the ECG remained unchanged at 60 bpm. Simultaneously, a sudden loss of power in all EEG frequencies on the spectral display of the BIS monitor (Figure 1) and a sudden drop in rSO_2_ from 75% to 52% (Figure 2) occurred, followed by a gradual decrease in ETCO_2_ to 20 mmHg (baseline ETCO_2_ between 35 and 45 mmHg). A non-palpable carotid pulse and an intra-operative CA with Pulseless Electrical Activity (PEA) were identified.

Advanced life support (ALS) was initiated with compressions in the knee-chest position. After the surgical incision dressing, the patient was moved to the supine position. Return of spontaneous circulation (ROSC) occurred after one cycle of ALS. NIBP recovered to a MAP of 60-70 mmHg, capnography returned to baseline, and 100% SpO2 was achieved. A large rSO2 increase was concomitantly observed, with the return of bilateral DSA frequencies between 0 and 13Hz (Figures 1, 2).

After ROSC, ST-segment elevation above 4.5 mm at DII and V5 was observed, and a 12-lead ECG showed diffuse ST-segment elevation. A transthoracic echocardiogram (TTE) performed in the operation room demonstrated global hypokinesis with segmentary contractility changes and the patient was transferred to the cardiac laboratory catheterization.

Coronary arteries were normal, but ventriculography revealed hypercontractility of the basal segments with apical akinesia (“apical ballooning”) indicative of stress cardiomyopathy. ST-segment elevation reversed two hours after the onset of TTS clinical signs. Subsequently, a diagnosis of Takotsubo cardiomyopathy was made. A cerebral computed tomography was performed and ruled out subarachnoid hemorrhage.

The patient was transferred to an intensive care unit (ICU), under sedation and ventilation and she remained hemodynamically stable, without vasopressor requirements. Extubation was possible nine hours after admission. Afterward, the patient was asymptomatic, without neurological deficits, and denied awareness during the surgical procedure, resuscitation, or postoperatively. Her stay in the ICU was uneventful. Investigation during this period included a TTE that registered hypokinesia of the distal half of all left ventricle walls with moderate depression of the global systemic function of the left ventricle (LVEF estimated by the Simpson biplane method of +/- 37%). She was discharged to the ward three days after ICU admission.

Hospital discharge occurred eight days later. The surgical wound healed, no CSF leak occurred, and she could walk on the ward with only mild back pain. It was agreed to postpone surgery until further evaluation by the cardiology team.

## Discussion

We describe a case of intraoperative CA secondary to TTS, two hours into a spine surgery in a patient in the knee-chest position. Few cases of intraoperative TTS have been reported, the majority occurring following anesthesia induction, surgical incision, or a painful stimulus [3]. In our case, PEA as a manifestation of TTS occurred during the intraoperative period without any apparent stressful stimulus contrary to previously described cases.

PEA may be a challenging diagnosis and integration of several monitored parameters may be the key not to delaying the diagnosis [1]. Monitoring rSO2 has been previously described as a feasible tool during resuscitation as it can lead to earlier detection of CA and ROSC [8]. In our case, the sudden loss of bilateral EEG and decrease of cerebral rSO2 were in striking contrast with the presence of an unchanged ECG, and immediately sustained the possibility of a PEA. We believe this is the first report of the use of intraoperative neuromonitoring during a CA in PEA.

TTS typically affects postmenopausal women with emotional or physical stress factors. Severe anxiety and surgical procedure are sources of stress, that induce a stage of high serum catecholamines. This may be responsible for contractile dysfunction observed in TTS [2,3]. In our case, the stability of monitored parameters, namely those reflecting the hypnotic and anti-nociceptive components of anesthesia, led us to believe that inadequate anesthesia was not the cause of the TTS. It is characterized by ST elevations on the ECG and elevated cardiac biomarkers, with no signs of coronary disease [3]. Its diagnosis is typically supported by an echocardiogram, usually showing extensive hypokinesia/akinesia of the left ventricular mid segments and classic “apical ballooning” on the ventriculography [2]. In our case, the event was presented with all the characteristics described above.

Our patient was medicated with clomipramine, a tricyclic antidepressant that is a serotonin-norepinephrine reuptake inhibitor. This group of drugs has already been associated as a precipitating cause for TTS [9]. Although there have been no reports associating clomipramine with TTS, its long-term use may have contributed to an increased catecholaminergic state. The exact etiology and pathophysiology of TTS are not known yet, so the knowledge of precipitants' causes is essential.

## Conclusions

To our knowledge, this is the first reported case of CA secondary to TTS during general anesthesia in the knee-chest position. It shows that TTS can occur despite what seemed to be adequate anesthesia. It also illustrates the usefulness of multimodal monitoring with cerebral monitoring to assist in the prompt diagnosis of PEA. Cerebral monitoring contributed to improving the outcome of this severe condition showing its usefulness in surgeries where the brain is not directly at risk. Cerebral oxygenation and EEG spectral analysis are now widely available in operating rooms, they are non-invasive and easy to interpret, so their use deserves to be considered in cases other than carotid, cardiac or brain surgeries.
